# A comparison of ARMS and DNA sequencing for mutation analysis in clinical biopsy samples

**DOI:** 10.1186/1756-9966-29-132

**Published:** 2010-10-06

**Authors:** Gillian Ellison, Emma Donald, Gael McWalter, Lucy Knight, Lynn Fletcher, James Sherwood, Mireille Cantarini, Maria Orr, Georgina Speake

**Affiliations:** 1 AstraZeneca, Alderley Park, Macclesfield, Cheshire, SK10 4TG, UK; 2 Previous Address: AstraZeneca, Alderley Park, Macclesfield, Cheshire, SK10 4TG, UK; 3 Quintiles Global Oncology, Station House, Bracknell, Berkshire RG12 1HX, UK

## Abstract

**Background:**

We have compared mutation analysis by DNA sequencing and Amplification Refractory Mutation System™ (ARMS™) for their ability to detect mutations in clinical biopsy specimens.

**Methods:**

We have evaluated five real-time ARMS assays: *BRAF *1799T>A, [this includes V600E and V600K] and *NRAS *182A>G [Q61R] and 181C>A [Q61K] in melanoma, *EGFR *2573T>G [L858R], 2235-2249del15 [E746-A750del] in non-small-cell lung cancer, and compared the results to DNA sequencing of the mutation 'hot-spots' in these genes in formalin-fixed paraffin-embedded tumour (FF-PET) DNA.

**Results:**

The ARMS assays maximised the number of samples that could be analysed when both the quality and quantity of DNA was low, and improved both the sensitivity and speed of analysis compared with sequencing. ARMS was more robust with fewer reaction failures compared with sequencing and was more sensitive as it was able to detect functional mutations that were not detected by DNA sequencing. DNA sequencing was able to detect a small number of lower frequency recurrent mutations across the exons screened that were not interrogated using the specific ARMS assays in these studies.

**Conclusions:**

ARMS was more sensitive and robust at detecting defined somatic mutations than DNA sequencing on clinical samples where the predominant sample type was FF-PET.

## Introduction

The molecular analysis of tumours has become increasingly important in recent years, particularly to aid the choice of drug therapy [1,2]. Assays to evaluate clinical samples, particularly if the results are used to determine treatment regimens, need to be rapid, precise and specific. However, the processes used to prepare tumours for standard pathology analysis for diagnosis, while preserving the tissue architecture, have a detrimental effect on DNA and RNA. Formalin fixation and subsequent embedding in paraffin tends to fragment and cause adducts in the DNA that can make analysis challenging [3]. In addition, tumour specimens are heterogeneous. They can contain surrounding and infiltrating normal cells, and not all tumour cells are identical. Analysis methods must therefore also be sensitive.

DNA sequencing is one of the most widely used methods for analysing DNA and has been successfully used to analyse and detect mutations in DNA derived from formalin-fixed paraffin-embedded tumours (FF-PETs) for many years. It is a well-established method, widely available and relatively inexpensive to use [4,5] and can detect any mutation in the sequence being analysed. DNA sequencing is often quoted as the 'gold standard' for DNA sequence analysis [6]. However, sequencing is not exquisitely sensitive. A mutation must be present in approximately 20% of the sample to be readily detected [7,8]. Studies in colorectal cancer have found the percentage mutation in a tumour sample to be as low as 6%, significantly lower than sequencing is able to detect [9]. Given the heterogeneity of tumours [10] the percentage is possibly even lower in some tumour biopsy specimens.

We have extensive experience in the development and use of the allele specific polymerase chain reaction (PCR)-based method ARMS™ (Amplification Refractory Mutation System) [11]. These assays are sensitive, routinely being able to detect at least 1% mutant in a normal DNA background, and are quick and easy to use. This PCR-based method can be further enhanced by the ability to analyse the results in a real-time, closed-tube format by incorporating fluorescent probes such as TaqMan [12], Scorpions [13], Molecular Beacons [14] or intercalating fluorescent dyes such as Yo-Pro [15] or Sybr green [16], which eliminates PCR product contamination and reduces the time to generate results. They perform well on FF-PET-derived DNA and their sensitivity makes them ideal for the analysis of heterogeneous tumour samples. Unlike sequencing, ARMS assays only detect the mutations they were designed to interrogate. However, this could be considered an advantage in a clinical setting so that decisions on treatment or patient-outcome results are based only on known, clinically validated mutations.

We have evaluated three real-time ARMS assays in melanoma tumour samples: *BRAF *1799T>A [this includes V600E and V600K], *NRAS *182A>G [Q61R] and 181C>A [Q61K], and two real-time ARMS assays in non-small-cell lung cancer (NSCLC) samples: *EGFR *2573T>G [L858R] and 2235-2249del15 [E746-A750del], for the analyses FF-PET DNA and compared the results to DNA sequencing of the exons containing mutation hot-spots for these genes (*BRAF *exon 15, *NRAS *exon 1, *EGFR *exons 18-21). The data presented in this paper will demonstrate that ARMS is superior to sequencing in both sensitivity and robustness on a large and diverse set of clinical tumour samples making ARMS a suitable choice for the analysis of known, well-characterised mutations such as those found in *RAS*, *BRAF *and *EGFR *compared to DNA sequencing.

## Methods

### Samples

Unresectable American Joint Committee on Cancer Stage 3 or 4 malignant melanoma samples were obtained as part of a phase II, multi-centre, open-label, parallel-group, randomised study to compare the efficacy of selumetinib (AZD6244) versus temozolomide. Locally advanced or metastatic NSCLC samples were obtained as part of a double-blind, placebo-controlled, parallel-group, multicentre, randomised, phase III study (Iressa Survival Evaluation in Lung Cancer (ISEL)) trial [17]. All patients provided written informed consent; the trials were ethically approved and performed according to principles of good clinical practice.

### Sample processing

All samples underwent a haematoxylin and eosin pathology review to confirm the presence of tumour in the samples. The NSCLC samples were macro-dissected by scraping only the tumour area that had been selected by a pathologist. No enrichment by macro-dissection was performed on the melanoma samples. This was because the planned primary analysis method was ARMS and macro-dissection was thought unnecessary due to the sensitivity of the method. Genomic DNA was extracted from thin sections totalling 40 μm by digestion in proteinase K for 48 h, boiling in 5% chelex, phase-extracting in chloroform, ethanol-precipitating and resuspending in 100 μl water [18]. This method eliminated the need for a xylene de-waxing step, thus reducing potential tissue loss. The same extraction method was used for both sample sets.

NSCLC DNA samples were quantified by quantitative PCR using primers and probes specific to alpha-1 antitrypsin: forward control primer AGGACACCGAGGAAGAGGACTT; reverse control primer GGAATCACCTTCTGTCTTCATTT, control probe Cy5-CTGCLTPAZGAGGGGAA-Elle (L = LNA (locked nucleic acid) modified C, P = LNA G, Z = LNA T). All primers and probes were manufactured by Eurogenetec. The primers were 0.1 μM and TaqMan probes at 0.5 μM. PCR was performed at 95°C for 10 min, followed by 40 cycles of 94°C for 45 s, 60°C for 1 min and 72°C for 45 s in the MX3000 (Stratagene). Data were collected at the 60°C stage of the reaction. A dilution series of known amounts of normal genomic DNA (Roche) was amplified in the same machine run and the MX3000 software extrapolated the DNA concentration of the unknown samples from the standard curve generated.

This method of quantification was used rather than spectrophotometry as it only measures amplifiable DNA. Only NSCLC samples with detectable amplifiable DNA (>5 genomic copies/μl) were used for mutation analysis. Extracted melanoma DNA was not quantified prior to mutation analysis. Instead, the control reaction was used to determine DNA extraction success concurrent with the ARMS reactions. The assays were considered to have failed when the control reaction fell below the limits of detection. If the DNA was found to exceed the maximum recommended DNA amount, it was diluted below 1000 genomic copies per reaction and re-analysed.

DNA was extracted from 171 melanoma samples (158 were FF-PET and 13 were frozen) and 433 FF-PET NSCLC samples.

### ARMS analysis

Five microlitres of melanoma DNA diluted 1/5 in water (Sigma) was added to each mutation assay containing primers that specifically amplified either *BRAF *1799T>A (resulting in either V600E, V600K or V600D amino acid changes depending on the presence of an additional mutation at position 1798 or 1800) and *NRAS *181C>A and 182A>G (Q61R) mutations, and primers that amplify an unrelated sequence, which acts as a control for the presence of DNA. Brilliant Multiplex Q-PCR Master mix (Stratagene) was used and supplemented with bovine serum albumin (New England Biolabs) to reduce the PCR inhibitory effects of melanin in the melanoma samples. Assays were performed in duplicate.

The primer pairs and TaqMan probes were as follows: *BRAF *ARMS primer AAAAATAGGTGATTTTGGTCTAGCTACATA, reverse primer TAGTTGAGACCTTCAATGACTTTCTAGTAA, probe VIC-AATCTCGATGGAGTGGGTCCCATCAGTTTGAACA-TAMRA; *NRAS *Q61K ARMS primer GTTTGTTGGACATACTGGATACAGCTGGTA, reverse primer TTCCCCATAAAGATTCAGAACACAAAGATC, probe Yakima Yellow-ALATGAGGALAGGCGAAGGC-BHQ1; *NRAS *Q61R ARMS primer AZALTGGATACAGLTGGACP, reverse primer TTCCCCATAAAGATTCAGAACACAAAGATC, probe Yakima Yellow-ALATGAGGALAGGCGAAGGC-BHQ1, forward control primer AGGACACCGAGGAAGAGGACTT; reverse control primer GGAATCACCTTCTGTCTTCATTT, control probe Cy5-CTGCLTPAZGAGGGGAA-Elle (L = LNA (locked nucleic acid) modified C, P = LNA G, Z = LNA T). All primers and probes were manufactured by Eurogenetec. All ARMS primer pairs were at a concentration of 1 μM, the control reaction primers were 0.1 μM and TaqMan probes at 0.5 μM. PCR was performed at 95°C for 10 min, followed by 40 cycles of 94°C for 45 s, 60°C for 1 min and 72°C for 45 s in the MX3000 (Stratagene). Data were collected at the 60°C stage of the reaction. Cell line DNA admixtures containing the mutation of interest in a normal DNA background (ranging from 100% mutant - 1% mutant in a normal background) was amplified in the same machine runs to act as positive controls and evaluate limit of detection and sensitivity. A mutation positive result was only accepted if it was present in independent PCRs generated from the same DNA sample. Seven hundred nanograms of normal genomic DNA was used as a negative control to assess assay specificity. This amount of DNA was significantly greater than typical DNA yields from FF-PET material. Results were not designated positive unless the mutation was detected before any non-specificity to control for false positive results.

*EGFR *ARMS analyses were performed on the NSCLC DNA samples by DxS (Manchester) [17].

### DNA sequencing

*BRAF *and *NRAS *sequencing analysis were conducted on melanoma DNA samples only. Five microlitres of tumour DNA diluted 1/5 in water was added to triplicate PCR assays containing PCR buffer II at 2 mM MgCl_2_, 3.75 units AmpliTaqGOLD (ABI), 200 μM dNTP (ABgene) and supplemented with bovine serum albumin (New England Biolabs) with 5' end tagged primers (forward primer tag: ACTGTAAAACGACGGCCAGT; reverse primer tag: ACCAGGAAACAGCTATGACC) that amplified *BRAF *exon 15, and *NRAS *exon 2: *BRAF *exon 15 forward TTTCCTTTACTTACTACACCTC, reverse CTTTCTAGTAACTCAGCAGCATC; *NRAS *exon 2 forward CCCCCAGGATTCTTACAGAA; reverse ATACACAGAGGAAGCCTTCG. PCRs were conducted using the following cycling conditions: 95°C, 10 min, (94°C, 30 s, 58°C, 30 s, 72°C, 1 min) × 40 cycles, 72°C, 10 min.

*EGFR *analysis was conducted on NSCLC DNA samples. Five microlitres of tumour DNA diluted 1/5 in water was added to triplicate PCR assays containing PCR buffer II at 2 mM MgCl_2_, 3.75 units AmpliTaqGOLD (ABI), 200 μM dNTP (ABgene) and supplemented with bovine serum albumin (New England Biolabs) with 5' end tagged primers (forward primer tag: ACTGTAAAACGACGGCCAGT; reverse primer tag: ACCAGGAAACAGCTATGACC) that amplified *EGFR *exons 18 to 21: *EGFR *exon 18 forward CCTTCCAAATGAGCTGGCAAGTG, reverse TCTCACAGGACCACTGATTACTG; *EGFR *exon 19 forward GCAGCATGTGGCACCATCTCAC, reverse CAGGGTCTAGAGCAGAGCAGC; *EGFR *exon 20 forward CGCATTCATGCGTCTTCACCTG, reverse CTATCCCAGGAGCGCAGACCG; *EGFR *exon 21 forward TCGACGTGGAGAGGCTCAGAG and reverse CTGCGAGCTCACCCAGAATGTC. PCRs were conducted using the flowing conditions: 95°C 10 min, (94°C, 20 s, 61°C, 30 s (dropping 0.5°C/cycle), 72°C, 1 min) × 13 cycles, (94°C, 20 s, 57°C, 30 s, 72°C,1 min) × 30 cycles, 72°C, 10 min.

Resulting PCR products were bidirectionally sequenced using primers complimentary to the Forward and Reverse tags on the primary PCR primers using ABI Big Dye sequencing, and analysed using Mutation Surveyor software (SoftGenetics). To eliminate false positive mutations occurring due to sample fixation artefacts, a mutation result was only accepted if it was present in at least two out of three independent PCRs in at least one of each Forward and Reverse sequencing traces.

## Results

### Melanoma analysis

Out of the 177 melanoma samples extracted, 163 (92%) were successfully analysed by ARMS as indicated by the presence of the control reaction, and 156 (88%) were successfully analysed by DNA sequencing as indicated by readable sequencing traces.

In total, 69 *BRAF *mutations were detected using a combination of both methods; 67 of these were at codon 600, one at codon 601 (K601E) and another at codon 581 (N581S). The 67 codon 600 mutations (1799T>A) were detected using the ARMS assay but only 46 of these were detected by DNA sequencing. Forty-one of these were V600E mutations and five were V600K. The *BRAF *1799T>A ARMS assay could detect V600E, V600K and V600D mutations as they all contain mutations at the same nucleotide position, but could not distinguish between them. The V600K and V600D amino acid changes contain additional nucleotide substitutions, although these do not affect the ARMS assay's ability to detect 1799T>A change. Ten samples were *BRAF *ARMS mutation positive but the mutation was not seen in the sequencing traces, demonstrating that ARMS was more sensitive than DNA sequencing. No sequencing data were obtained for 11 ARMS positive samples as they failed to amplify or give readable sequencing traces. The failure of DNA sequencing could in part be explained by the difference in size of the ARMS PCR product and the sequencing product that were 179 base pairs (bp) and 212 bp, respectively. The sequencing product was longer to encompass the whole exon. There were no *BRAF *1799T>A mutations detected by DNA sequencing that were not detected by ARMS although DNA sequencing revealed two mutations in different codons that could not be detected by the ARMS assay. *BRAF *mutations found in the melanoma samples using a combination of DNA sequencing and ARMS are listed in Table 1.

**Table 1:** *BRAF *mutations found in the melanoma samples using a combination of DNA sequencing and ARMS.

Mutation	No. of mutations	Detected by ARMS	Detected by sequencing
V600E, V600K (1799T > A)	67	67	46
K601E	1	ND	1
N581S	1	ND	1
Total	69	67	48

ND, not detectable.

In total, 28 *NRAS *mutations were detected using a combination of both methods. Twelve were 182A>G (Q61R), 15 were 181C>A (Q61K) and one 37G>C (G13R). The G13R mutation was not detectable by the specific ARMS assays used. Twenty-seven were detected using the ARMS assay whereas only 21 (including the G13R mutation) were detected by DNA sequencing. Of the 27 ARMS mutation positive samples, three were sequencing negative and four failed sequencing. The failure of DNA sequencing was not due to a size difference between the ARMS PCR products (190 and 201 bp) and the sequencing product (140 bp) as the sequencing product was smaller in this case. There were no *NRAS *181C>A and 182A>G 1799T>A mutations detected by DNA sequencing that were not detected by ARMS.

*NRAS *mutations found in the melanoma samples using a combination of DNA sequencing and ARMS are listed in Table 2.

**Table 2:** *NRAS *mutations found in the melanoma samples using a combination of DNA sequencing and ARMS.

Mutation	No. of mutations	Detected by ARMS	Detected by sequencing
G13R	1	ND	1
Q61R	12	12	10
Q61K	15	15	10
Total	28	27	21

ND, not detectable.

#### Performance on low-quality FF-PET DNA

All the frozen samples amplified well in both assays. 158 samples were FF-PET. Sixteen samples failed to generate ARMS assay data (i.e. no control reaction detected) and 25 failed to generate sequencing data due to low DNA amounts. Nine of these samples failed both sequencing and ARMS, 7 samples failed ARMS only, and 16 samples failed sequencing only. Eleven samples that failed sequencing were found to be *BRAF *ARMS positive.

**Figure 1:**
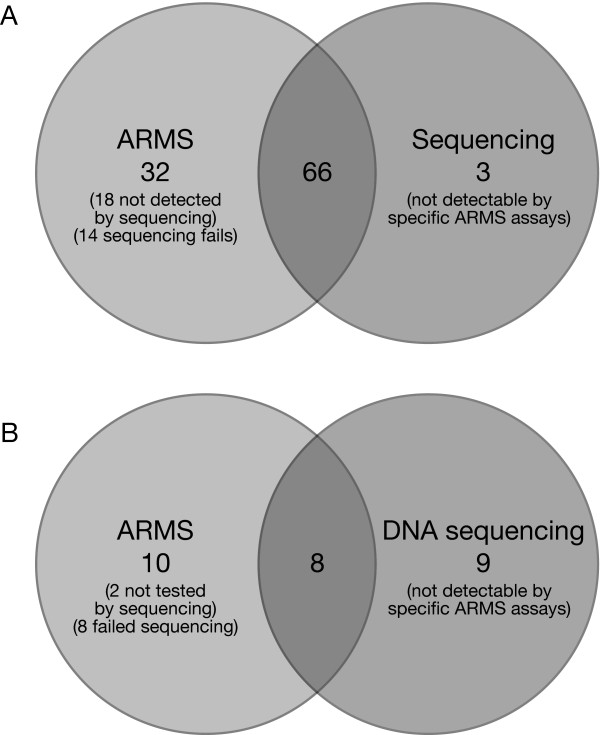
**(A) Melanoma mutations**. Sixty-six *BRAF *or *NRAS *mutations were detected in the melanoma samples by both methods. ARMS detected an additional 32 mutations. Eighteen of these were not detected on the sequencing traces and 14 failed to sequence. Three mutations were detected by sequencing only. These were mutations that the ARMS assays were not designed to detect. (B) NSCLC mutations. Eight *EGFR *mutations were detected in the NSCLC samples by both methods. ARMS detected an additional 10 mutations. Two of these were not analysed by sequencing as the DNA amount was too low and eight failed to sequence. Nine mutations were detected by sequencing only. These were mutations that the ARMS assays were not designed to detect. Note that there were 27 mutations in 26 patients as one sample was found to contain two mutations.

These data indicate that ARMS is more successful at genotyping samples in low quality FF-PET extracted DNA. The results are summarised in Fig. 1A.

#### DNA quantity and ability to detect mutations

**Figure 2:**
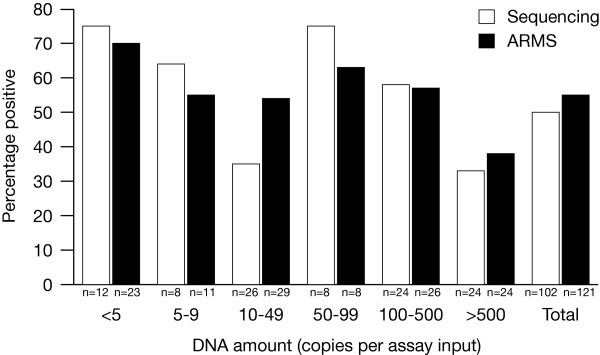
**Mutation detection success on varying the amount of input DNA**. The DNA yield was grouped into categories and the percentage of mutations detected calculated for each group. The n values are the successful number of sequencing and ARMS analyses. The lower yielding samples did not show any decrease in the numbers of *BRAF *or *NRAS *mutations detected. Both DNA sequencing and ARMS gave similar results in each category although overall ARMS detected more mutations. As the DNA concentration increased the number of successful sequencing reactions also increased: at >50 copies per assay input, the analysis success rate was very similar for both ARMS and sequencing.

The first 121 of the melanoma samples yielding DNA were grouped by DNA yield to determine if at low DNA quantity the ability to detect mutations was reduced. The groupings (>5 copies, 5-9 copies, 10-49 copies, 50-99 copies, 100-500 copies and >500 copies) were based on the amount of DNA in the control reactions that could be used to estimate the amount of DNA in the sample. There were more groupings at the lower DNA concentration as it was thought that any effect would be more likely to be observed in these samples. There was no decrease in the ability to detect mutations as the DNA amount decreased. Both DNA sequencing and ARMS gave similar results in each category although overall ARMS detected more mutations. As the DNA concentration increased the number of successful sequencing reactions also increased: at >50 copies per assay input, the analysis success rate was very similar for both ARMS and sequencing. The results are shown in Fig. 2.

In some samples at high DNA concentrations (>1000 copies assay input) non-specific signal did occur in the ARMS. In these samples it was important to dilute DNA below 1000 copies per assay input and repeat the analysis. This only affected a minority of samples - most samples in excess of this DNA limit did not exhibit any non-specificity at all. Why this should occur in some samples and not others is not known but adds to the difficulty of analysing FF-PET DNA.

### Non-small-cell lung cancer analysis

Of the 433 samples extracted, 215 yielded detectable amounts of DNA greater than 5 copies/μl. This was lower than the melanoma samples and was probably due to the age of the samples and also no guidance on sample collection was given as mutation analysis was not initially planned for these samples. We compared DNA sequencing success rate to DNA amount of the first 100 samples into the NSCLC study that yielded detectable DNA. We found that below 10 copies there was a 90% failure rate to either amplify or generate readable sequencing traces. Between 10 and 40 copies the success rate was 25%. As the DNA concentration improved, the success rate also improved. At this point we decided only to analyse samples by sequencing that were greater than 10 copies/μl, and performed a nested PCR to improve the success rate on the 10-40 copies/μl samples. Eighteen of the 215 samples yielded very low DNA amounts (5-10 genomic copies/μl). This was insufficient for sequencing and these samples were only analysed by ARMS. Of these 18 samples, two also failed ARMS analysis.

Twenty-six mutation-positive patients were identified using both methods and the mutations detected are described in Table 3. One patient was found to have both an exon 19 deletion (del L747-P753 ins Q) by sequencing only and an L858R point mutation by ARMS only.

**Table 3:** *EGFR *mutations found in the NSCLC samples using a combination of DNA sequencing and ARMS.

Mutation	No. of mutations	Detected by ARMS*	Detected by sequencing
del E746-A750	9	9	4
del E749-E758insQP	1	ND	1
del L747-P753 ins Q	1*	ND	1
del E749-A753 ins P	1	ND	1
del L747-P753 ins S	1	ND	1
Other deletions	1	ND	1
G719A	1	ND	1
A743S	1	ND	1
L858R	9*	9	4
L861Q	2	ND	2
Total	27	18	17

EGFR-2 kit detecting L858R and del E746-A750 only. Other mutations not detectable by this version of the kit.

*One patient had both an exon 19 deletion and an L858R point mutation.ND, not detectable.

Nine mutations were neither L858R nor del E746-A750 and could only be detected by sequencing. Ten mutations were detected by ARMS but not sequencing. Of these, two were from the 18 samples not analysed by sequencing due to low DNA yield and eight were in samples which failed to sequence. The failure of DNA sequencing could in part be explained by the difference in size of the ARMS PCR products and the sequencing products. Although the ARMS assay details were proprietary it was believed that the PCR products were less than 150 bp, whereas the sequencing products ranged from 291-511 bp. When DNA sequence data were obtained the mutation status matched that generated by ARMS. The results are summarised in Fig. 1B.

## Discussion

In this study ARMS has been found to be both more sensitive and robust at detecting somatic mutations in clinical material than DNA sequencing. There were no examples where ARMS did not detect an assay-specific mutation that was detected by DNA sequencing. There were 42 mutations detected by ARMS that were not detected by DNA sequencing either due to low quantity or quality DNA causing assay fails or low mutant DNA compared to normal DNA in the sample that was beyond the detection limit of sequencing. They were not believed to be false positive results as they were known mutations, the results were reproducible and adequate controls were analysed in parallel. There were 12 mutations detected by sequencing that were not detected by ARMS because the ARMS assays used were not designed to detect these mutations, either because the mutations were rare (melanoma study) or ARMS assays had not yet been developed to detect these mutations. However, using the larger panel of ARMS assays now available the number of mutations detected by ARMS would be significantly increased with potentially only 1 mutation being missed from this study.

Even though ARMS is the more sensitive technique, in the NSCLC samples from which DNA sequence could be obtained no mutations were detected by ARMS that were not detected by sequencing. Mutations were only missed by DNA sequencing due to assay fails owing to the low amounts of poor quality, fragmented DNA yielded from the samples. This probably reflected the fact that these samples had been macro-dissected prior to analysis, enriching for tumour and increasing the abundance of mutant DNA in the sample. However, the macro-dissection process was very time-consuming and labour-intensive and required specialist pathologist input. Reducing the size of the PCR amplicons used in sequencing may also have reduced the number of samples that failed in DNA sequencing.

In the melanoma study, no macro-dissection was performed. This was because the planned primary analysis method was ARMS and macro-dissection was thought unnecessary due to the sensitivity of the method. The results of the melanoma analysis reflected this as not all mutations detected by ARMS were visible on sequencing traces. They were not believed to be false positive results as they were known mutations, the results were reproducible and high levels of normal DNA was used as a control for non-specificity.

As the analysis method for the melanoma study was ARMS we did not quantify the DNA prior to analysis because the ARMS assays contained a control reaction that could be used to semi-quantify the DNA at the same time as performing the diagnostic reaction. Eliminating the quantification step reduced the analysis time. For the NSCLC study, however, the primary method was sequencing as there were only two *EGFR *mutant ARMS assays available at the time of the study and while the common mutations were well established, the number of rarer mutations being discovered was still increasing.

To reduce the effort of sequencing in the many samples (179 samples were >10 copies/μl [empirically determined cut-off for sequencing]) that would have failed in 90% of the cases and to reduce the costs of the commercial assays we quantified the extracted DNA and only analysed the samples where there was a good chance of success. For NSCLC *EGFR *ARMS analysis we only analysed DNA samples greater than five copies of DNA/5 μl (with 5 μl added to the PCR) as this was the limit of detection claimed for these assays (218 samples were <5 copies/μl).

Interestingly, as the melanoma DNA yield decreased, there was little drop-off in the percentage of *BRAF *or *NRAS *mutations detected using either ARMS or sequencing. This would suggest that even at low DNA assay input the samples were representative of the tumours and that at low DNA input there were probably few, if any, false negative results. Analysing all samples was a good strategy to maximise the numbers of mutations detected in this study set where 88% of the samples yielded detectable DNA.

In a research setting one of the strengths of sequencing is that it detects unknown mutations as well as known ones. However, in a clinical setting it is likely that decisions will be made on the basis of known characterised mutations. When analysing genes where mutations are found clustered in one or two exons, like *KRAS*, much less DNA is required for sequencing than for ARMS, although this can be reduced by multiplexing ARMS reactions. This can be an advantage when only very small biopsies with low DNA are available. Sequencing also offers an advantage when genes contain many mutations throughout the coding region, such as *p53*, *BRCA *and *APC*. To develop the potentially hundreds of individual mutation detection assays required would be extremely time-consuming and require positive mutation controls to show that the assays are functioning correctly. Sequencing reactions tend to be easier to develop and standard genomic DNA is an adequate control.

It was important when performing sequencing that at least two independent PCRs were performed from the original genomic DNA to eliminate false positive errors. We were able to distinguish true mutations from artefactual mutations by only accepting mutations detected in at least two amplicons in forward and reverse sequencing directions. Approximately 2% of the exons sequenced contained an artefact. These were most commonly detected in samples with low DNA, probably because they were not masked by more abundant unaltered DNA. These artefacts are presumably caused by damage to the DNA during fixation in formalin. None of the artefacts found in singleton were known mutations. They were not reproducible in any subsequent PCR from the original DNA samples and we were unable to validate them using other mutation discovery methods including denaturing high-performance liquid chromatography, and cloning and sequencing. ARMS appeared to be less affected by DNA artefacts as the assays only targeted known mutations. Pathology information was also taken into account as this could often explain why mutations were present at a low level in a sample.

In contrast to the advantages of sequencing, the ARMS assays were easy to perform and interpret and lend themselves to better standardisation, which helps when performing mutation analysis on a global scale or as a companion diagnostic. As ARMS is very sensitive, routinely being able to detect at least 1% mutant in a background of normal DNA, this may reduce the need for macro-dissection which eliminates a labour-intensive, time-consuming step in the analysis process. By coupling ARMS with real-time PCR product detection the analysis process is further shortened as PCR products do not have to be processed, for example by agarose gel electrophoresis, and PCR product contamination is eliminated as reaction tubes do not need to be opened after the experiment is complete.

As ARMS is sensitive it can also be used on samples where the tumour content is very low, for example circulating free (cf) tumour DNA shed from the tumour into the blood [19,20] and in cytology samples [21,22]. This can be an advantage when a tumour sample is not available, for example if the tumour is inoperable or so badly processed that no DNA is extractable. However, in our experience, the mutation detection rates using alternative sources of tumour such as cf DNA tend to be lower than from a tumour biopsy.

In this study we have evaluated ARMS and DNA sequencing only; however, there are a growing number of alternative methods being established that may merit evaluation. All methods have their own merits and are chosen according to the task e.g. clinical trial methodology may be different to those employed in the diagnostic setting for sensitivity, cost, availability and a variety of other reasons. Test choice will differ as tests evolve and it is important to keep abreast of all available methods.

In our experience, ARMS is more sensitive and robust at detecting defined somatic mutations than DNA sequencing on clinical samples where the predominant sample type was FF-PET. Future developments in the field of mutation detection will be followed with anticipation as such technologies will be key to support personalised healthcare approaches that select patients for targeted treatments based on tumour mutation results.

## Competing interests

GE, ED, GM, LF, JS, MC, MO and GS are employees and shareholders of AstraZeneca. LK is a former employee of AstraZeneca and has no additional competing interests to declare.

## Authors' contributions

GE carried out the molecular genetic studies and drafted the manuscript. ED, GM, LK, LF and JS carried out the molecular analysis. MC, MO and GS participated in the design and coordination of the study. JM drafted the manuscript. All authors reviewed the draft manuscript and read and approved the final version for submission.
